# A Statistical Framework for Assessing the Relationship between Biomarkers and Clinical Endpoints in Alzheimer’s Disease

**DOI:** 10.14283/jpad.2024.126

**Published:** 2024-07-02

**Authors:** Tianle Chen, R. M. Hutchison, C. Rubel, J. Murphy, J. Xie, P. Montenigro, W. Cheng, K. Fraser, G. Dent, S. Hendrix, O. Hansson, P. Aisen, Y. Tian, J. O’Gorman

**Affiliations:** 1 Biogen Inc., 225 Binney St., Cambridge, MA 02142 USA; 2 Pentara Corporation, Millcreek, UT USA; 3 Clinical Memory Research Unit, Department of Clinical Sciences, Lund University, Malmö, Sweden; 4 Memory Clinic, Skåne University Hospital, Malmö, Sweden; 5 Alzheimer’s Therapeutic Research Institute, University of Southern California, San Diego, CA USA

**Keywords:** Alzheimer’s disease, biomarkers, statistics, plasma p-tau, positron emission tomography

## Abstract

Changes in biomarker levels of Alzheimer’s disease (AD) reflect underlying pathophysiological changes in the brain and can provide evidence of direct and downstream treatment effects linked to disease modification. Recent results from clinical trials of anti–amyloid β (Aβ) treatments have raised the question of how to best characterize the relationship between AD biomarkers and clinical endpoints. Consensus methodology for assessing such relationships is lacking, leading to inconsistent evaluation and reporting. In this review, we provide a statistical framework for reporting treatment effects on early and late accelerating AD biomarkers and assessing their relationship with clinical endpoints at the subject and group levels. Amyloid positron emission tomography (PET), plasma p-tau, and tau PET follow specific trajectories during AD and are used as exemplar cases to contrast biomarkers with early and late progression. Subject-level correlation was assessed using change from baseline in biomarkers versus change from baseline in clinical endpoints, and interpretation of the correlation is dependent on the biomarker and disease stage. Group-level correlation was assessed using the placebo-adjusted treatment effects on biomarkers versus those on clinical endpoints in each trial. This correlation leverages the fundamental advantages of randomized placebo-controlled trials and assesses the predictivity of a treatment effect on a biomarker or clinical benefit. Harmonization in the assessment of treatment effects on biomarkers and their relationship to clinical endpoints will provide a wealth of comparable data across clinical trials and may yield new insights for the treatment of AD.

## Introduction

**A**lzheimer’s disease (AD) is a complex, multifactorial neurodegenerative disease (1, 2). Changes in the measured levels of biological markers of AD (ie, AD biomarkers) reflect underlying pathophysiological changes in the brain that occur well before clinical symptoms are present and progress over decades in a continuous manner (1–4). Measurement of AD biomarkers can assist in clinical diagnosis, demonstrate target engagement during a clinical trial, provide supportive evidence of disease modification, and predict future disease progression (5, 6). Biomarker changes in response to treatment with an investigational therapeutic agent may provide evidence of clinical efficacy and/or suggest downstream effects that may be linked to disease modification (5, 7, 8). However, AD biomarkers mirror the complexity of the disease process with specific characteristics and trajectories, and the statistical analysis of AD biomarkers requires understanding of the biology of AD, the dynamics of AD biomarkers, and application of appropriate statistical methodologies. In the past 20 years, more than 200 candidate therapeutic agents for AD have failed or been abandoned due to safety concerns or a lack of clinical efficacy (9). However, the current AD clinical development landscape includes dozens of therapeutic agents that target the underlying pathophysiology of AD, with the goal of significantly altering disease progression (10, 11). In particular, the accumulating data from a class of second-generation anti–amyloid β (Aβ) monoclonal antibodies (11–14) have augmented the need to define best practices for detecting and reporting treatment effects on AD biomarkers and their relationships with clinical endpoints within and across clinical development programs. Moreover, the availability of large and comprehensive AD biomarker datasets has allowed the field to interrogate new prognostic relationships between endpoints and may guide future trial design.

Consensus methodology for analysis and presentation of biomarker data is lacking across the AD field, in particular during assessment of the relationship between biomarker and clinical endpoints. Selection of correlational analyses differ across sponsors and regulatory reviewers, which can lead to inconsistent evaluation and reporting. In some programs, the participants treated with placebo and those receiving active treatment are pooled when the relationship between biomarker and clinical endpoints is assessed, while sometimes this relationship is assessed in each active treatment arm separately or even in a subset of a treatment arm. Heterogeneity in statistical methodology can be due to 1) the lack of statistical consideration of the distinct features of biomarkers at different disease stages and 2) a lack of clarity of the specific research question to be answered. For example, is the analysis intending to demonstrate a prognostic relationship between two endpoints that is unrelated to treatment? Or does it intend to examine whether a treatment effect on a biomarker predicts a clinical benefit? One analysis might be appropriate for a biomarker that manifests concurrently with clinical symptoms but might not be appropriate for a biomarker with progression that began decades before disease onset.

In this review, we propose recommendations to accommodate the features of different types of biomarkers at different stages of AD with the appropriate statistical analysis. We also elucidate the distinctions between the correlational analyses at the subject level and group level and provide the recommended applications of each analysis. Amyloid positron emission tomography (PET), plasma p-tau, and tau PET data from clinical trials of AD were examined to elucidate biomarker trajectories. Recommendations for reporting treatment effects and for assessing the relationship between biomarkers and clinical measures at the subject level and group level are presented.

## Methodology

### Defining Classes of AD Biomarkers in a Statistical Framework

**Figure 1:**
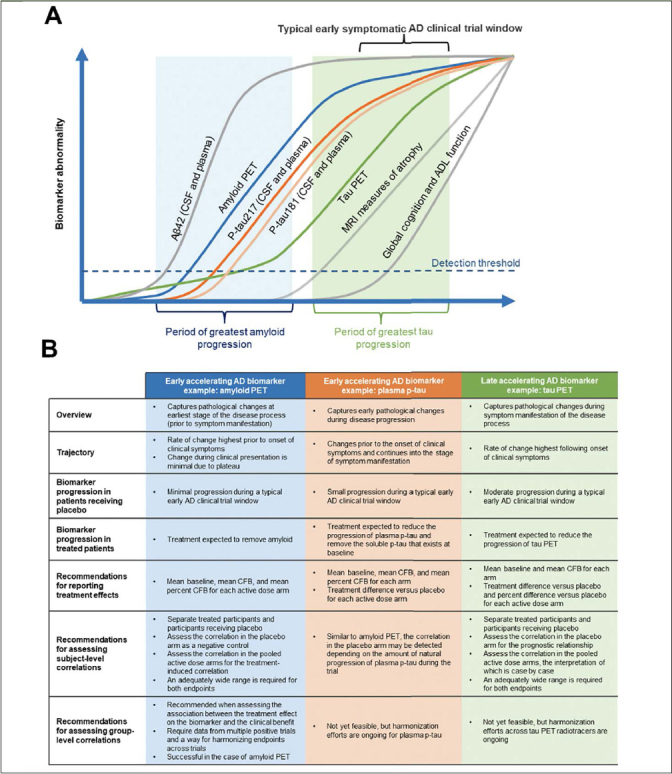
Trajectory, characteristics, and analysis recommendations for early and late accelerating AD biomarkers Figure adapted from Hansson O. Nat Med 2021;27:954-963. Aβ, amyloid beta; AD, Alzheimer’s disease; ADL, activities of daily living; CFB, change from baseline; CL, centiloid; CSF, cerebrospinal fluid; MRI, magnetic resonance imaging; PET, positron emission tomography; p-tau, phosphorylated tau.

Each AD biomarker exhibits a specific spatiotemporal pattern during disease progression, reflecting different disease pathologies and/or stages of disease (2–4). In this review, we have defined pathology as structural and cellular changes associated with AD and pathophysiology as functional disturbances and mechanisms underlying AD processes (15). Under these definitions, tau PET is a measure of tau pathology and would also be defined as a measure of tau pathophysiology along with other tau biomarkers (ie, plasma p-tau) that do not measure structural or cellular changes associated with AD. While biomarker frameworks such as the ATX(N) system have been described and implemented in clinical research (16, 17), the focus of the current work is to define a statistical framework that guides interdisciplinary clinical trial design and analyses. For the purpose of this review and the statistical framework discussed, AD biomarkers can be broadly organized into two classes based on their temporal window of change during disease progression. Early accelerating AD biomarkers (eg, cerebrospinal fluid [CSF]/plasma Aβ42, amyloid PET, and plasma p-tau) are those that capture pathophysiological changes at an early stage of the disease process, many years before clinical symptoms are present (3). Late accelerating AD biomarkers (eg, volumetric magnetic resonance imagining [MRI] and tau PET) reflect pathophysiological changes that are temporally close to clinical symptom manifestation (3). Figure 1A presents the changes in early versus late accelerating biomarkers over time. It should be noted that a binary classification is used for simplicity and to illustrate the biomarker classes and characteristics in this review. There are overlaps between the biomarker classes discussed that may require special considerations.

There is evidence of Aβ accumulation decades prior to clinical symptoms in AD (18). Biomarker confirmation of Aβ pathology is routinely used to complement a clinical diagnosis, and the advent of Aβ PET imaging has enabled noninvasive visualization of fibrillar or insoluble Aβ plaques (19). Moreover, in clinical trials of AD, Aβ PET is now commonly used as a screening tool and a biological endpoint (14, 20, 21). Thus, biomarker measures of accumulated Aβ in the brain (eg, amyloid PET imaging and CSF and plasma Aβ levels) reflect pathological changes at the earliest stages of the disease process prior to symptom manifestation (Figure 1A and 1B) (22). The rate of change for amyloid PET, an early accelerating AD biomarker, is highest prior to the onset of clinical symptoms (22). During the clinical manifestation phase, detection of change is limited as accumulation has plateaued (3, 22, 23). In the context of a clinical study of an Aβ removing treatment (14), minimal progression of an early accelerating biomarker is anticipated in the placebo group, compared with an expected significant reduction from baseline for patients receiving active treatment.

The presence of Aβ plaques in the brain is believed to drive downstream tau pathology (15, 24, 25). In AD, the highly soluble tau protein forms intracellular fibrillar structures of aggregated, post-translationally modified neurofibrillary tangles that are associated with synaptic and neuronal loss (19, 24). Accumulation of tau, as assessed by tau PET, marks the later stages of AD, with tau tangles following a spatiotemporal pattern of spread starting with transentorhinal, limbic, and finally neocortical regions (17, 26). A growing body of evidence confirms that tau PET imaging is a powerful tool for visualizing and quantifying tau pathology, allowing non-invasive observation of the spatial distribution and temporal changes in tau aggregates (27). As a late accelerating AD biomarker, tau PET can capture pathological changes during the symptomatic phase of disease (Figure 1B), with the highest rate of change associated temporally with the onset and exacerbation of clinical symptoms (3, 28, 29). In the context of a clinical study of an effective investigational agent in early symptomatic AD with a mechanism of action that slows progression, one would expect brain region–specific progression of tau PET in the placebo group compared with reduced progression in the treatment group.

Various neuropathology, Aβ, and tau PET studies have shown that changes in the levels of soluble phosphorylated p-tau are linked to accumulation of both Aβ plaques, during early disease stages, and tau tangles, during later disease stages (30). Consistent with the hypothesis that Aβ pathology drives tau accumulation, evidence suggests that soluble p-tau levels may mediate the effect of Aβ fibrils on the formation of pathological tau aggregates (3, 31–33). Assays have been developed to detect several species of p-tau in plasma, including p-tau181, p-tau217, and p-tau231, and can differentiate AD from other neurodegenerative diseases with high accuracy (3, 34). Although there are likely differences between p-tau species in the time course of changes across the AD continuum, in general as another example of an early accelerating biomarker, plasma p-tau levels reflect early pathophysiological changes of disease progression (Figure 1B) (3), with changes detected prior to the onset of clinical symptoms with a continued increase as clinical symptoms manifest (3, 35, 36). As it is later than amyloid PET in the disease progression cascade (3), plasma p-tau demonstrates modest progression in participants receiving placebo during a typical early symptomatic AD clinical trial window in contrast to the minimal progression in amyloid PET. A treatment effect on plasma p-tau would be expected to reduce soluble p-tau levels in plasma below the levels that exist at baseline (14, 35).

The distinct temporal and spatial trajectory of each AD biomarker during a clinical trial highlights the nuances of assessing the relationship between specific biomarkers and clinical measures and the necessity of using the most appropriate statistical approaches that align with the specific characteristics of each biomarker.

The statistical framework and considerations provided here using examples from each biomarker class can be applied to other biomarkers. They may also be applied to biomarkers in other disease areas to guide clinical trial design and data analysis.

### Trial Data Sources

Data from the clinical trials of several second-generation anti Aβ monoclonal antibodies were used for illustrative purposes in this review. Data from two Phase 3 clinical trials of aducanumab (EMERGE and ENGAGE) (14, 37, 38), the lecanemab Phase 3 CLARITY trial (12, 39), and the gantenerumab Phase 3 trials GRADUATE I and II (40-42) were used as examples for recommendations for reporting treatment effects on amyloid PET, plasma p-tau181, and tau PET.

Data from the Phase 3 EMERGE trial of aducanumab and hypothetical tau PET data were used to illustrate recommendations for performing subject-level correlations between biomarkers and clinical endpoints. Data from the Phase 1b (PRIME) and Phase 3 (EMERGE and ENGAGE) trials of aducanumab (14, 43, 44), the lecanemab Phase 2 and Phase 3 (CLARITY AD) trials (12, 45, 46), the gantenerumab GRADUATE I and II Phase 3 trials (40), and the donanemab TRAILBLAZER-ALZ and TRAILBLAZER-ALZ 2 (13, 47–49) Phase 2 and 3 clinical trials were used as examples of recommendations for performing group-level correlations between treatment effects on amyloid PET and Clinical Dementia Rating Scale-Sum of Boxes (CDR-SB) scores.

### Subject- and Group-Level Correlation Analyses

Correlations were analyzed in two ways. Subject-level correlations were assessed using each participant’s change from baseline (CFB) for the biomarker versus CFB on the clinical endpoint during a given AD trial and were adjusted for baseline covariates as needed. The CFB is a single delta representing the difference between post-baseline and baseline for a certain endpoint. Group-level correlation was assessed using the placebo-adjusted treatment effects on the biomarker versus that on the clinical endpoint in each active dose arm. The placebo-adjusted treatment effect represents the difference in CFB between the 2 groups (ie, double delta).

### The Centiloid Scale for Standardizing Reporting of Results Across Amyloid PET Tracers

Standardized uptake value ratio (SUVR) is a semiquantitative method for analyzing and normalizing amyloid PET images and is dependent on the radiotracer, target, and reference regions; analysis methodology; and data acquisition method used (50, 51). Deviations in methodology lead to challenges in quantitatively evaluating amyloid PET results across studies or between clinical programs (50). Klunk et al. developed a scale of centiloid (CL) units to standardize reporting of amyloid PET data using a post hoc linear transformation (50, 52). A CL value of 0 represents the population mean level of Aβ-negative individuals, and 100 represents the population mean level of Aβ burden in individuals with mild to moderate severity dementia due to AD (52). The CL scale, which translates amyloid PET results into units that can be compared and pooled across tracers and clinical trials, is being increasingly used across the field.

## Results

### Recommendations for Reporting Treatment Effects on Biomarkers

#### Reporting Treatment Effects for Early Accelerating AD Biomarkers

**Figure 2:**
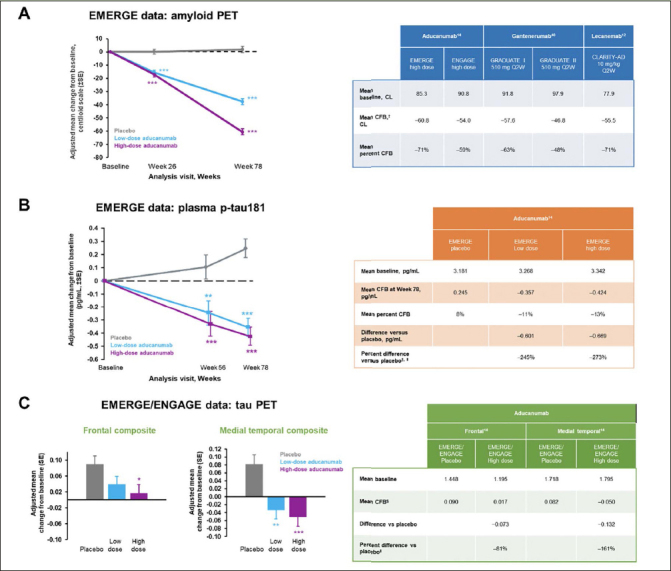
Recommendations for reporting of treatment effects on biomarkers * p<0.05, ** p<0.01, *** p<0.001. Figures adapted from Budd Haeberlein S, et al. J Prev Alzheimers Dis 2022;9(2):197-210. Creative Commons Attribution 4.0 International License (https://creativecommons.org/licenses/by/4.0/). ^+^ Mean CFB in amyloid PET was reported at 24 months for GRADUATE I and II and at 18 months for the other trials. * Percent difference versus placebo is included here to illustrate the impact of a small change in the denominator on the percent difference. We do not recommend reporting this value for plasma p-tau. § Mean CFB for tau PET was reported at the post-baseline visit with a mean duration of 14 months. ∥ Percent difference versus placebo is the difference in mean CFB between active dose and placebo arms divided by the CFB in placebo. CL, centiloid; CFB, change from baseline; PET, positron emission tomography; p-tau, phosphorylated tau; SE, standard error; Q2W, once every 2 weeks.

Data from clinical trials of aducanumab, gantenerumab, and lecanemab were used in Figure 2A to illustrate recommendations for reporting treatment effects on early accelerating biomarkers such as amyloid PET when using the CL scale. We recommend presenting the estimated mean baseline value and the mean CFB in each active dose arm from the statistical models specified in the trial, as well as the corresponding mean percent CFB, calculated as the mean CFB divided by the mean baseline value in each arm (Figure 2A, table). We also advise presenting the measure of variability corresponding to each estimate as appropriate (eg, standard error and the 95% confidence interval for the mean CFB). The mean percent CFB introduced in this review is calculated using group mean-level estimates. It is distinct from the subject-level percent change that has been used in other analyses. The CL scale facilitates a meaningful interpretation of the mean CFB when reported as a percentage, as it anchors the population mean of Aβ-negative individuals to 0, which in theory eliminates the offset. Therefore, the mean percent CFB represents the relative amount of Aβ plaques removed compared with the total amount of plaques that exceeds the threshold for Aβ negativity at baseline. If amyloid PET results are reported using the SUVR scale, then we do not recommend reporting the mean percent CFB since the SUVR scale, as a ratio, has an offset of around 1 (these values may differ slightly depending on which radiotracer is used). Although CL is a linear transformation of SUVR, without the rescaling step to eliminate the offset, which is part of the denominator, the percent CFB calculated on the SUVR scale is much smaller than that on the CL scale and does not carry a meaningful interpretation of the “amount of Aβ removed relative to total”. Furthermore, the CL scale allows for amyloid PET scans collected during clinical trials and using multiple tracers to be pooled rather than separated into subcohorts (52).

Aβ positivity is part of the inclusion criteria for current early symptomatic AD clinical trials when Aβ accumulation has hit a plateau and there is minimal progression during the trial (14). Thus, the CFB of amyloid PET in the placebo arm is negligible, and the treatment difference of the active dose arm versus placebo is effectively close to CFB by design. The CFB in placebo and treatment difference versus placebo can be presented for comprehensiveness and as confirmation of the expected result for participants in the placebo arm. The percent difference versus placebo is calculated as the treatment difference in mean CFB between treatment versus placebo, divided by the mean CFB in the placebo arm, and is commonly reported for clinical endpoints in AD (13, 14). This statistic is not applicable to amyloid PET because the denominator is close to zero due to the plateau of Aβ accumulation during the trial in participants at this stage of AD. The percent difference applies more appropriately to biomarkers and clinical endpoints that show moderate progression during a clinical trial, such as tau PET and clinical endpoints for which the treatment is intended to slow their progression. This will be explained in the next section.

As seen during the EMERGE trial (Figure 2B), natural progression of plasma p-tau can be observed in the placebo arm, but the change is relatively small in magnitude. For plasma p-tau, we recommend presenting the mean baseline and the estimated mean CFB for the placebo and active dose arms, the corresponding mean percent CFB for each arm, and the treatment difference versus placebo. The mean percent CFB here can be useful for a within-study comparison, but there are limitations to its generalization and interpretation. No CL-like scale exists for plasma p-tau; thus, there is no scale to anchor the negative threshold to 0, and the mean percent CFB does not directly link to the relative amount of change in pathology. Similar to amyloid PET, we do not recommend reporting the percent difference versus placebo for plasma p-tau. Since the denominator (plasma p-tau progression in the placebo arm) is very small compared with the numerator (the difference in mean CFB between the placebo and active dose arms), a small variation in the denominator can cause large variations in the percent difference versus placebo. For example, with a denominator of 0.245 (which corresponds to an 8% increase relative to baseline in the placebo group), the percent difference versus placebo for plasma p-tau in the EMERGE high-dose arm was -273% (Figure 2B). If the denominator was 0.160 (which corresponds to a 5% increase relative to baseline in the placebo group), the percent difference would be -365%. Thus, a 3% decrease in the denominator would lead to a 92% increase in the percent difference versus placebo.

#### Reporting Treatment Effects for Late Accelerating AD Biomarkers

As a late accelerating biomarker, tau continues to accumulate over the course of a typical early symptomatic AD trial (3) (Figure 1B); depending on the therapeutic agent and the specific brain region, the tau PET signal can be slowed, stopped, or even reduced below baseline levels (14, 53). While a universal CL-like scale for tau PET harmonization is not yet available, recent efforts using a joint propagation model to establish tau PET units of harmonization termed “CenTauR” appear promising (54, 55). Treatment effects on tau PET may be brain region dependent; thus, regions of interest should be prespecified in the analysis. Examples of data from EMERGE and ENGAGE are presented in Figure 2C to illustrate the different magnitude of treatment effect on tau PET signal in the discrete, a priori-defined brain composites analyzed (14). When treatment effects on tau PET are reported, we advise presenting the mean baseline, the estimated mean CFBs for both the placebo arm and the active dose arms, the treatment difference versus placebo, and the percent difference versus placebo in each active dose arm. The percent difference versus placebo represents the relative amount of tau signal reduced by the treatment compared with the amount accumulated during natural disease progression in the placebo arm. It is possible for the magnitude of the percent difference to be >100% if the tau PET signal is reduced below baseline by the treatment.

### *Recommendations for Assessing Subject-Level Correlations*

#### Early Accelerating AD Biomarker Considerations for Subject-Level Correlations

**Figure 3:**
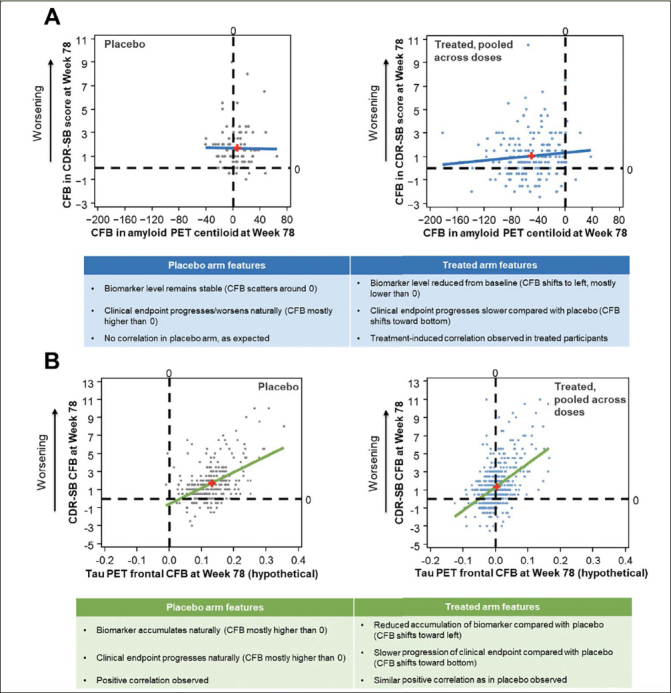
Subject-level correlation between aducanumab EMERGE amyloid PET or hypothetical tau PET and clinical endpoints Least-squares regression lines are displayed and red plus signs indicate mean x,y values. CDR-SB, Clinical Dementia Rating–Sum of Boxes; CFB, change from baseline; PET, positron emission tomography.

When assessing subject-level correlations between an early accelerating biomarker and clinical endpoints, such as amyloid PET and CDR-SB, we advise separating the treated participants and participants receiving placebo so that the correlation in the placebo arm can be assessed as a negative control. The examination of a wide range of CFBs on both endpoints results in better evaluation of the correlation and should be assessed in the pooled active dose arms. As an example, data from EMERGE are presented in Figure 3A to illustrate subject-level correlations between CFB in CDR-SB and amyloid PET. In the placebo arm of EMERGE, amyloid PET levels remained stable and the CFB was scattered around 0. CDR-SB progressed naturally during the trial, and the CFB was mostly higher than 0 (positive CFB in CDR-SB represents clinical worsening). There was no correlation between amyloid PET and clinical progression in the placebo arm, as expected. In contrast, in the pooled active dose arms, amyloid PET levels were reduced from baseline, with the CFB shifting to the left and mostly lower than 0. Clinical endpoint progression in the active dose arms was slower compared with that in the placebo arm, and the CFB shifted toward the bottom of the plot. Thus, a treatment-induced correlation was observed in treated participants in EMERGE.

While the treatment-induced correlation does provide evidence of association between the treatment effect on Aβ, such as a reduction in the amount of Aβ plaques, and the clinical benefit, such as slowing of clinical progression, there are some caveats. The true correlation might be obscured due to the narrow range of CFBs, participant heterogenicity, and large variation in clinical measures. For example, inclusion of only one dose level in a Phase 2 clinical trial can lead to a narrow range of CFBs and limit exploration of the subject-level correlation. In addition, it can also provide less value to a group-level meta-analysis as there is no way to adjust for the study and/or compound-level variation. Furthermore, combining placebo and treated participants is generally not recommended as the prognostic relationship confounds the correlation under treatment. On the other hand, determining the correlation in each active dose arm separately (when there are multiple active dose arms in the study) or even determining the correlation in a subset of participants within one dose arm is not recommended as this leads to a very narrow range of CFBs and limits the assessment of the correlation.

Although plasma p-tau becomes abnormal later in the disease continuum compared with amyloid PET, plasma p-tau is another example of an early accelerating biomarker for which changes significantly precede the onset of clinical symptoms (3). In the typical early symptomatic AD trial, we expect to see relatively small progression in plasma p-tau levels, and an effective treatment is expected to reduce soluble p-tau levels in plasma below the levels that exist at baseline (Figure 1). Therefore, the statistical considerations for assessing subject-level correlations of plasma p-tau with clinical endpoints share similar features as those of amyloid PET: the correlation should be assessed separately in participants receiving placebo and participants receiving active treatment. In the placebo arm, we do not expect to see a correlation between plasma p-tau and clinical endpoints, but this is not as definite for Aβ PET, for which progression can be negligible compared with p-tau. A correlation may be detected, depending on the amount of natural progression of plasma p-tau during the trial and the sensitivity of the plasma p-tau measure to detect small changes. In the pooled active dose arms, we expect to see a treatment-induced correlation if the treatment has an effect on both reducing soluble plasma p-tau below levels at baseline and slowing clinical progression. In the placebo arms of the aducanumab Phase 3 studies (14), no correlations between plasma p-tau181 and the clinical endpoints were observed, except for Alzheimer’s Disease Assessment Scale Cognitive Subscale 13 score in ENGAGE. In the pooled active dose arms of EMERGE and ENGAGE, a greater reduction in plasma p-tau181 levels was associated with less clinical progression across all four clinical measures, indicative of a treatment-induced correlation (Table 1).

**Table 1:** Subject-level correlation between plasma p-tau and clinical endpoints

**Association between change in p-tau181 and efficacy at Week 78**		**Hypothesized correlation**	**Correlation (p value)**	
			**EMERGE (n=521-528)**	**ENGAGE (n=584-588)**
Plasma p-tau181	CDR-SB	Positive	0.11 (p=0.0137)	0.15 (p=0.0002)
	MMSE	Negative	−0.20 (p&lt;0.0001)	−0.16 (p=0.0001)
	ADAS-Cog 13	Positive	0.16 (p=0.0003)	0.15 (p=0.0002)
	ADCS-ADL MCI	Negative	−0.12 (p=0.0056)	−0.13 (p=0.0013)

Table adapted from Budd Haeberlein S, et al. J Prev Alzheimers Dis 2022;9(2):197–210. Creative Commons Attribution 4.0 International License (https://creativecommons.org/licenses/by/4.0/). Correlations are partial Spearman correlations assessed in pooled low- and high-dose aducanumab-treated groups, adjusted for baseline p-tau, baseline clinical endpoint, and age. ADAS-Cog 13, Alzheimer’s Disease Assessment Scale Cognitive Subscale; ADCS-ADL-MCI, Alzheimer’s Disease Cooperative Study Activities of Daily Living Inventory (mild cognitive impairment version); CDR-SB, Clinical Dementia Rating–Sum of Boxes; MMSE, Mini-Mental State Examination; p-tau, phosphorylated tau.

#### Late Accelerating AD Biomarker Considerations for Subject-Level Correlations

When subject-level correlations are assessed between late accelerating biomarkers and clinical endpoints, such as tau PET and CDR-SB, correlational analyses should be performed in the placebo arm first to assess the prognostic relationship, which is not associated with active treatment, and then in the pooled active dose arms to assess the association in CFBs after exposure to treatment (Figure 3B). The treatment may affect tau levels and clinical endpoints differently and could dissociate their prognostic relationship in various ways. Moreover, an adequately wide range of CFBs in both the biomarker and clinical endpoints is required to properly assess the subject-level correlation.

Hypothetical data are presented in Figure 3B to illustrate a possible outcome of the subject-level correlation between later biomarkers such as tau PET and clinical endpoints. In this case, we modeled the treatment as having an effect on both tau PET and CDR-SB during the trial. In the placebo arm, the biomarker accumulated and the clinical endpoint progressed during the course of a clinical trial. The CFB is mostly higher than 0 for both measures, and a positive correlation is observed between the biomarker and the clinical endpoint CFB values. In the pooled active dose arms, a reduced accumulation of the biomarker compared with that in the placebo arm is observed, and the CFB shifts toward the left. In participants receiving active treatment, there is slower progression of the clinical endpoint compared with participants receiving placebo; the CFB shifts toward the bottom of the plot, and a positive correlation is observed, similar to what is seen in the placebo group. Another possible outcome could be that the treatment has no effect on either endpoint, yet a positive correlation is observed in both the active treated and placebo arms. A third possibility is that the treatment may have a different magnitude of effect on the biomarker versus the clinical endpoint. For example, if the treatment stops the accumulation of the biomarker during the trial, leading to a CFB around 0, then although it also leads to attenuation of clinical progression, the correlation may not be observed due to the narrow range of the CFB in the biomarker measurement. Therefore, careful examination of the specific biomarker features and the probable outcome of the investigational therapeutic agent is needed when planning and interpreting the results of subject-level correlation analysis.

As discussed earlier, subject-level correlation has many caveats (56) and is thus generally not conclusive when the association is assessed between the effect of treatment on late accelerating biomarkers and clinical endpoints. If a correlation is observed during a clinical trial between the CFBs of two endpoints that are temporally related in their trajectory, the correlation may exist as a purely prognostic relationship independent of treatment. An example of this phenomenon is the subject-level correlation observed between volumetric MRI and clinical data from the Phase 3 trials of bapineuzumab (57). Volumetric MRI is a late accelerating biomarker similar to tau PET. Bapineuzumab failed to show a treatment effect on the rate of whole-brain atrophy and clinical endpoints, but a correlation was observed between clinical progression and brain atrophy at Week 78 in both participants receiving placebo and those receiving active treatment (57). Thus, the correlation observed suggests that brain atrophy is likely a prognostic biomarker for clinical progression, but in this example, it may not be related to treatment effect. Other groups have expanded on the caveats that should be considered when assessing the relationship between the biomarker and the clinical outcome (58). Please note that the temporal relationship mentioned in this paper refers to the broader temporal relationship across the entire disease spectrum. The narrower temporal relationship within a clinical trial (eg, a biomarker measure at 12 months vs clinical endpoints at 18 months) is not discussed in this review.

While subject-level correlation can be used to assess the relationship between biomarkers and clinical endpoints, the interpretation varies among biomarkers at different stages. For early accelerating biomarkers, the treatment-induced correlation provides evidence of association between the treatment effect on the biomarker and the clinical endpoint. For later accelerating biomarkers, various situations are possible and should be assessed on a case-by-case basis. Observing a subject-level correlation is neither necessary nor sufficient for demonstrating therapeutic benefit. Furthermore, subject-level correlation does not demonstrate the ability of the biomarker to predict the clinical benefit (eg, the treatment effect of a clinically relevant outcome) (59). Moreover, there are other possible limitations, such as a narrow range of CFBs, participant heterogeneity, and large variation in both clinical measures and biomarkers, which may obscure the true correlation.

### *Recommendations for Assessing Group-Level Correlations*

If the research question is to assess the association between the treatment effect on a specific biomarker and the clinical benefit, group-level correlation is an appropriate approach. Group-level correlation was assessed using the placebo-adjusted treatment effects on the biomarker (ie, double delta) versus that on the clinical endpoint in each active dose arm. This approach leverages the fundamental advantages of randomized, placebo-controlled trials in that 1) they directly assess the association between the placebo-adjusted treatment benefits on biomarkers versus clinical measures, aligning with the definition of a surrogate endpoint that predicts clinical benefit (59) and 2) participant heterogeneity is addressed by randomization in a randomized clinical trial, and large variation in clinical measures is addressed by the use of model-adjusted group means. Group-level correlation has wide applications in principle and has been used in many disease areas, including oncology, cardiovascular disease, and HIV (60). In AD, it applies to biomarkers at all stages (early or late) regardless of their trajectories. It also applies to different types of study endpoints such as continuous, categorical, and time to event.

**Figure 4:**
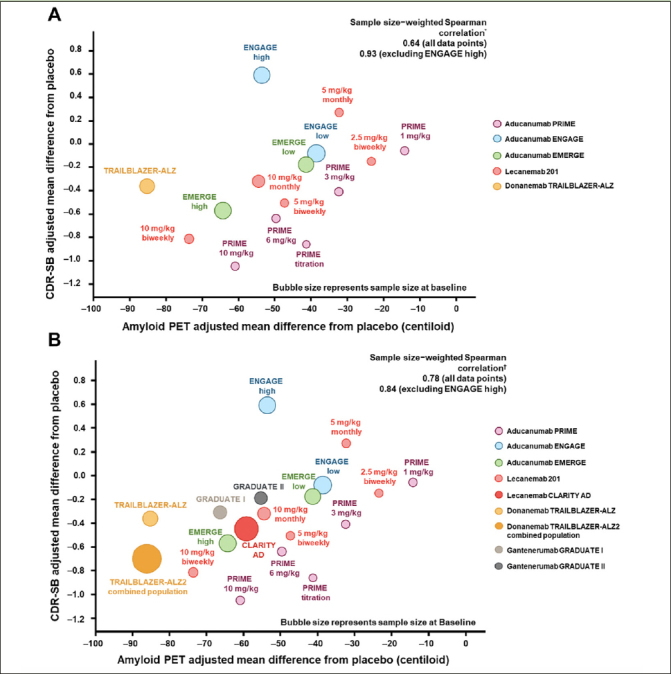
Group-level correlations: treatment effects on Aβ and CDR-SB in second-generation Aβ monoclonal antibodies in 2021 and 2023 (12–14, 45, 47) Figures adapted from Budd Haeberlein S, et al. J Prev Alzheimers Dis 2022;9(2):197-210. Creative Commons Attribution 4.0 International License (https://creativecommons.org/licenses/by/4.0/). * Sample size–weighted partial Spearman correlations adjusted for study indicator of aducanumab PRIME, aducanumab Phase 3, donanemab, and lecanemab. † Sample size–weighted partial Spearman correlations adjusted for study indicator of aducanumab PRIME, aducanumab Phase 3, donanemab, lecanemab, and gantenerumab. Sample size and clinical results are based on the subpopulation with amyloid PET assessments, except for lecanemab CLARITY AD and gantenerumab GRADUATE I and II, in which intention-to-treat clinical results were used due to availability. Aβ, amyloid β; CDR-SB, Clinical Dementia Rating Scale–Sum of Boxes; PET, positron emission tomography.

Group-level correlation is a meta-analysis; thus, it requires a number of clinical trials demonstrating treatment effects on both the biomarker and the clinical endpoints for accurate examination of the correlation. As an example, the emergence of recent data from multiple clinical trials of anti-Aβ antibodies in early symptomatic AD (12, 13, 40, 47) has provided sufficient data for group-level correlation across therapeutic agents with a similar mechanism of action. In 2021, with the available data at the time, the group-level correlation between treatment effects on amyloid PET and CDR-SB provided supportive evidence that reduction in the amount of Aβ plaques was associated with clinical benefit (Figure 4A). Zhu et al. (61) from the US Food and Drug Administration performed a similar group-level analysis that included second-generation and earlier generations of monoclonal anti-Aβ antibodies for which minimal to no change in Aβ PET was observed. Other groups have assessed the correlation between Aβ plaque removal by second-generation anti-Aβ mAbs measured by amyloid PET and CDR-SB relative reduction using meta-regressions (62). Group-level correlations using data from clinical trials of second-generation anti-Aβ mAbs up to the year 2023 are presented in Figure 4B. This addition of emerging data that support the clinical-biomarker relationship lowers the probability of a false-positive finding and increases confidence in this association for the field. Furthermore, the use of the CL scale allows for cross-program meta-analyses. Group-level correlation is powerful in Aβ PET analysis because of the use of the CL scale and the availability of Aβ PET and clinical data from multiple clinical trials of AD.

The interpretation of disease-related imaging markers, and comparability across different trials and imaging modalities, is greatly improved when standardized outcome measures are defined. Group-level correlation across multiple programs for plasma p-tau and tau PET is not yet feasible. This is due to the lack of a common scale to harmonize biomarker results across studies (54) and the lack of clinical trials that demonstrate a treatment effect on both the biomarker and the clinical endpoints.

Preliminary efforts supporting development and adoption of a universal scale for tau PET quantification are ongoing and appear promising (54, 63). A harmonization workstream is currently underway for tau PET, which may facilitate group-level meta-analysis across different compounds. The HEAD study is a multicenter longitudinal clinical trial that aims to compare and standardize cross-sectional and longitudinal tau tangle measurements obtained with the tau PET tracers flortaucipir and MK-6240 (64). The objective of the HEAD study is to elucidate the advantages and caveats of tau PET tracers in clinical trials and provide parameters to integrate their estimates (64). The Critical Path Institute is also leading a working group to standardize a scale for quantification of tau PET tracers across radiopharmaceuticals (63).

The different species of plasma p-tau (eg, 217 vs 181), different measurement platforms, lack of harmonization across assay methods and standardization of sample pre-analytical procedures, and different ways of data transformation used (original scale vs log scale for analysis) make it difficult to compare results across studies. Ongoing harmonization and standardization efforts such as those underway by the Standardization of Alzheimer’s Blood Biomarkers Program of the Global Biomarker Standardization Consortium (65) and continued understanding of the biological differences between p-tau species and their respective longitudinal trajectories will facilitate future interpretation.

## Conclusion

In this work, we classified AD biomarkers into early accelerating and late accelerating classes based on their trajectories during AD progression and introduced their corresponding features. We provided statistical recommendations on reporting treatment effects and assessing the relationship between biomarkers and clinical endpoints for each class of biomarkers by using amyloid PET, plasma p-tau, and tau PET data from recent early symptomatic AD clinical trials as exemplars. The interpretation for subject-level correlation varies across biomarkers and their trajectories and requires case-by-case examinations. Group-level correlation is instrumental in assessing the association between the treatment effect on the biomarker and the clinical benefit and has wide generalizability across diseases. It is important to understand each biomarker’s features and apply the appropriate correlation analysis that addresses the selected research questions. The recommendations provided in this review apply a wholistic approach to assessment of the relationship between biomarkers and clinical measures in which the temporal and spatial trajectory of each biomarker is considered.

The statistical framework described in this review applies to clinical trials of early symptomatic AD. In preclinical trials, these recommendations will likely shift, as the field further defines biomarker trajectories in cognitively unimpaired at-risk individuals, defined as individuals with the presence of early accelerating biomarkers (ie, plasma and CSF Aβ, Aβ PET) who are at risk of cognitive impairment associated with AD and those with subjective cognitive decline (66). The AHEAD 3–45 trial (NCT04468659) is evaluating lecanemab treatment in preclinical AD, which is defined as the stage at which cortical accumulation of Aβ is present before manifestation of clinical impairment (21). AHEAD 3–45 uses both early accelerating (plasma and CSF Aβ, Aβ PET) and late accelerating (tau PET) biomarkers to evaluate whether lecanemab can slow the accumulation of tau and prevent the cognitive decline associated with preclinical AD (21). The availability of approved Aβ-lowering treatments for symptomatic disease may result in an increase in informative attrition for those who progress in the trial; thus, adjustment of the analysis plan or study design itself may be required. The results of AHEAD 3–45 and other trials examining preclinical AD, including the recently completed A4 study (67) and the ongoing TRAILBLAZER ALZ-3 trial (NCT05026866) (68), will expand the field’s understanding of the spatiotemporal pattern between biomarkers and the appropriate statistical considerations in preclinical stages of AD.

Correlations between AD biomarkers are not in the scope of this review; however, there is evidence that combinations of biomarker profiles may be more sensitive correlates of clinical status at specific disease stages (69) and might support surrogacy. Additional research is needed to confirm if biomarker profiles can better differentiate stages of AD when they are incorporated into prognostic models rather than when they are assessed individually (69).

Harmonization across clinical trial programs in the reporting and assessment of treatment effects on biomarkers and clinical endpoints will provide a wealth of data that may yield new insights for the treatment of AD. This harmonization should not impinge on scientific exploration of the study data but will provide a common language in which the results generated can further advance trial design and execution and ultimately help improve treatment outcomes in patients with AD.
